# Granulomatous lymphocytic interstitial lung disease in common variable immune deficiency: an in-depth clinical, immunological, functional and radiological exploration with a focus on its management, challenged by chronic CMV infection

**DOI:** 10.3389/fimmu.2025.1589052

**Published:** 2025-05-15

**Authors:** Mattia Moratti, Gioacchino Schifino, Francesco Baccelli, Simona Ferrari, Elisabetta Magrini, Mirna Bassi, Aldo Guerrieri, Maurizio Zompatori, Marcello Lanari, Francesca Conti

**Affiliations:** ^1^ Specialty School of Paediatrics, University of Bologna, Bologna, Italy; ^2^ Department of Biomedicine and Prevention, Molecular Medicine and Applied Biotechnology, University of Rome Tor Vergata, Rome, Italy; ^3^ Unità Operativa di Pneumologia - Clinica delle Malattie dell’apparato respiratorio - Arcispedale Sant’Anna – Azienda Ospedaliero Universitaria di Ferrara, Ferrara, Italy; ^4^ Pediatric Hematology and Oncology, Istituto di Ricovero e Cura a Carattere Scientifico (IRCCS) Azienda Ospedaliero-Universitaria di Bologna, Bologna, Italy; ^5^ Department of Medical and Surgical Sciences (DIMEC), University of Bologna, Bologna, Italy; ^6^ Genetic Unit, Istituto di Ricovero e Cura a Carattere Scientifico (IRCCS) Azienda Ospedaliero-Universitaria di Bologna, Bologna, Italy; ^7^ Laboratory of Immuno-Haematology-Laboratorio Unico Metropolitano, Azienda Unità Sanitaria Locale (USL), Bologna, Italy; ^8^ Respiratory and Critical Care Unit, Istituto di Ricovero e Cura a Carattere Scientifico (IRCCS) Azienda Ospedaliero Universitaria di Bologna, Bologna, Italy; ^9^ Radiologia, Villa Erbosa, Gruppo San Donato, Bologna, Italy; ^10^ Pediatric Unit, Istituto di Ricovero e Cura a Carattere Scientifico (IRCCS) Azienda Ospedaliero-Universitaria di Bologna, Bologna, Italy

**Keywords:** chronic CMV, CVID, GLILD, immune-dysregulation, immunological biomarkers, immunophenotype, MMF, rituximab

## Abstract

**Background:**

Common variable immune deficiency (CVID) is the most prevalent inborn error of immunity (IEI), marked by diverse clinical-immunological phenotypes and significant immune-dysregulation, including granulomatous lymphocytic interstitial lung disease (GLILD). GLILD is a severe manifestation of CVID, contributing to reduced life expectancy and a challenging diagnosis due to its insidious and non-specific clinical course. Current management strategies for GLILD rely on expert opinion due to a lack of randomized controlled trials (RCTs).

**Objectives:**

This study aims to provide a comprehensive immunophenotypical characterization of CVID patients with and without GLILD, investigate predictive biomarkers for GLILD development, and explore therapeutic strategies, particularly during concomitant SARS-CoV-2 and chronic cytomegalovirus (CMV) infections.

**Sources:**

Primary data were collected from a cohort of 25 patients with CVID who underwent high-resolution computed tomography (HRCT), immunophenotyping, and serum immunoglobulin analysis at diagnosis and after immunoglobulin replacement therapy. Existing literature on CVID and GLILD biomarkers, immunological profiles, and therapeutic interventions informed comparative analyses.

**Content:**

Patients with GLILD exhibited distinct immunophenotypical features, including reduced regulatory T-cells, CD8+ naïve, central memory T-cells, and B-cell subsets (memory and switched memory), alongside increased CD21low B-cells and naïve B-cells, indicative of chronic inflammation-driven immune activation. IgA and IgG4 concentrations were significantly lower in patients with GLILD at diagnosis. Immunosuppressive therapy, predominantly mycophenolate mofetil (MMF), demonstrated favorable clinical and functional outcomes, though radiological progression persisted in some cases. CMV infection in patients with GLILD on immunosuppressants resulted in favorable outcomes, underscoring the importance of personalized treatment strategies.

**Implications:**

This study highlights novel immunological markers and clinical-radiological patterns as potential predictors for GLILD, advocating for their integration into diagnostic and monitoring frameworks to reduce reliance on invasive histopathology. Future research should focus on validating biomarkers and conducting RCTs to establish evidence-based guidelines for GLILD management.

## 1 Introduction

Common variable immune deficiency (CVID) is the most prevalent clinically significant inborn error of immunity (IEI) (1, 2), with an incidence of up to 1 in 10,000 individuals (3), accounting for approximately 40% of IEI cases in the USA (4). CVID encompasses a spectrum of clinical and immunological phenotypes, and in nearly 70% of cases, no single causative gene defect is identified (2, 5, 6).

Currently, Chapel’s CVID classification recognizes four main phenotypes with distinct clinical courses and prognostic significance: (1) absence of disease-related complications (previously termed the “infection only” phenotype), (2) immune cytopenias, (3) polyclonal lymphoproliferation, and (4) persistent unexplained enteropathy (7, 8).

Infection susceptibility remains a hallmark of CVID, affecting 70% to 99% of patients, particularly in the respiratory tract due to pyogenic bacteria (9–11). However, with the widespread adoption of immunoglobulin replacement therapy (IgRT) as the first-line immunoprophylactic treatment, the primary causes of CVID-related morbidity and mortality have shifted from infections to immune dysregulation, manifesting as autoimmunity, hyperinflammation, allergy, enteropathy, lymphoproliferation, and malignancy (12).

Patients with immune-dysregulation have an 11-fold higher risk of mortality compared to those with the “infection only” phenotype (13), and this risk is not mitigated by IgRT dosage (14). Notably, more than 70% of CVID patients exhibit immune dysregulation, with immune cytopenias, polyclonal lymphoproliferation, and non-infectious enteropathy affecting approximately 24%, 17%, and up to 40% of individuals, respectively (2, 9, 15). Despite its clinical significance, there is no consensus on the optimal management of CVID-related immune dysregulation, including the type, duration, and target population for immunosuppressive treatments (15).

Among immune dysregulation features, granulomatous lymphocytic interstitial lung disease (GLILD) represents a pulmonary manifestation of systemic dysimmunity, characterized by polyclonal lymphoproliferation (16). The Delphi consensus has defined GLILD as a distinct clinical-radiopathological interstitial lung disease (ILD) associated with lymphocytic and/or granulomatous lung infiltrates, unrelated to other pathological conditions (17). GLILD in CVID patients varies between 9% and 30%, and it may represent an initial presentation of the disease (18, 19). Importantly, GLILD is associated with a significant reduction in life expectancy, with a 50% decrease in survival after diagnosis, shortening the median lifespan from 28.9 to 13.7 years (12, 18).

Diagnosing GLILD is challenging, requiring a combination of clinical, radiological, and histopathological criteria (20, 21). The disease may be asymptomatic or present with non-specific symptoms, which do not reliably correlate with disease severity or lung function impairment (22, 23). Additionally, a standardized high-resolution computed tomography (HRCT) scoring system is lacking, further complicating early detection (24, 25). While histopathological confirmation remains the gold standard, as it requires invasive procedures such as transbronchial or video-assisted thoracoscopic (VATS) biopsy, this carries a non-negligible risk of morbidity and mortality (26).

Given the poor prognosis of GLILD, often exacerbated by delayed diagnosis and the absence of standardized treatment protocols, there is an urgent need to identify independent clinical, pulmonary functional, and imaging predictors of GLILD in patients with CVID. Several potential biomarkers have been associated with GLILD, including immune thrombocytopenia (ITP), autoimmune hemolytic anemia (AIHA), lymphadenopathy, splenomegaly, hepatosplenic lymphatic hyperplasia, high Baumann’s GLILD score, and reduced total lung capacity (TLC) and diffusing capacity for carbon monoxide (DLCO) (22, 27–29). However, little is known about immunological predictors, except for reductions in absolute CD8+ T cells, CD19+ B cells, switched-memory B cells, and marginal zone B cells, alongside an increase in CD21low B cells and decreased absolute IgG and IgA concentrations (22, 29).

Currently, GLILD management lacks evidence-based guidelines, and existing recommendations for glucocorticoids as first-line therapy and T-/B-cell-targeting immunosuppressants as second-line therapy are based solely on expert opinions in the absence of randomized controlled trials (RCTs) (17, 20).

In light of these challenges, we conducted a comprehensive immunophenotypic characterization of patients with CVID with and without GLILD, providing an in-depth analysis of humoral and cellular immune profiles. Additionally, we aimed to identify novel clinical, immunological, and radiological biomarkers that could predict GLILD development, potentially reducing the need for invasive histopathological confirmation. Finally, we explored the radiological, functional, immunological, and histopathological pulmonary features of GLILD and non-GLILD CVID patients, offering unique insights into therapeutic strategies, particularly in the context of SARS-CoV-2 and chronic cytomegalovirus (CMV) infection.

## 2 Materials and methods

This observational case-control study included 25 patients with CVID who were referred to the Immunology Service of the Pediatric Unit, IRCCS-Azienda Ospedaliero-Universitaria di Bologna, between October 2018 and December 2023.

CVID diagnosis was made following the European Society for Immunodeficiencies (ESID) registry working definitions (30).

The CVID cohort was divided into two main groups, GLILD subjects and controls, differing in the presence/absence of a previously diagnosed GLILD.

We executed a systematic comparison between the two groups through an exhaustive report of clinical and laboratory data.

All patients provided written informed consent to participate in this study, carried out according to the Declaration of Helsinki.

Recruitment was performed during regular medical check-ups in accordance with clinical practice. Biological samples, lung function tests (LFTs), and radiological imaging were obtained during diagnostic procedures after informed consent, without exposing patients to further check-ups and sampling exclusively for research.

### 2.1 Inclusion and exclusion criteria

The inclusion criteria for the GLILD group were the following: 1) diagnosis ascertained by a chest HRCT consistent with GLILD, a bronchoalveolar lavage (BAL) ruling out infectious pneumonia and/or colonization, and histological features suggestive for GLILD and inconsistent with malignancy at lung biopsy; 2) age >18 years at the time of GLILD diagnosis.

The final GLILD diagnosis was assessed by a multidisciplinary team involving an immunologist, a pneumonologist, a thoracic radiologist, and a pathologist, according to the British Lung Foundation/United Kingdom Primary Immunodeficiency Network Consensus Statement (17).

Controls were recruited from patients with CVID who were referred to the abovementioned Immunology Service for a periodic follow-up.

The inclusion criteria for the control group were the following: 1) a CVID clinical diagnosis according to ESID criteria in the absence of GLILD, reliably excluded through accurate clinical, immunological, functional, radiological, and histological assessments; 2) age- and sex-matching with the GLILD group; 3) age >18 years at the time of enrollment.

Exclusion criteria for the GLILD group included the presence of any clinical suspicion of a chronic active pulmonary infectious disease or lymphoma at GLILD diagnosis and age <18 years at time of GLILD diagnosis. The same criteria was applied for the control group without GLILD.

### 2.2 Demographic, clinical and focused pulmonary assessments

The main items considered were the following:

- Demographics (sex, age, date of CVID diagnosis, date of last follow-up);
- Immune-dysregulation affecting respiratory, gastrointestinal, endocrine, integumentary, osteoarticular, hematopoietic, cardiovascular, and urinary systems (organ-specific autoimmunity/hyper-inflammation/allergy, enteropathy, non-malignant lymphoproliferation, and malignancy);
- Revised Chapel’s CVID classification (8);
- Infectious phenotype (respiratory infections, IgRT route of administration and dosage, chemoprophylaxis, and splenectomy);
- Pulmonary phenotype (smoking status, professional exposures, ILD signs and symptoms, and therapeutic pharmacological and/or physiotherapeutic approaches).
- A lung HRCT, analyzed by a dedicated thoracic radiologist and pneumonologist, with attention to GLILD features according to Baumann’s method (25);
- LFTs, including forced expiratory volume in 1 second (FEV1), forced vital capacity (FVC), DLCO, and basal dyspnea according to the Modified Medical Research Council (mMRC) scale;
- Chemico-physical, immunological, and culture analyses of BAL;
- VATS and/or transbronchial biopsy, performed only in patients with a clinical-radiological-immunological-functional-based suspicion of GLILD.
- Therapeutic management (steroids, non-biological/biological immunosuppressants, chemoprophylaxis with trimethoprim/sulfamethoxazole, inflammatory modulation with macrolides, and respiratory physiokinesis therapy).

### 2.3 Laboratory investigations

A wide characterization of the cohort’s immunological characteristics was performed, including leukocyte formula (Sysmex XN-20) (31), an extended lymphocyte typization of both T-cell and B-cell compartments through multiparametric flow cytometry (32), and serum immunoglobulin concentration detection (IgG and subclasses, IgA, IgM, and IgE) with an immunoturbidimetric method (33).

More details regarding laboratory methods are provided in the **Supplementary Material**.

In order to avoid possible alterations attributable to immune system activation and to guarantee the same setting of immunophenotyping in the two groups, immunological data at CVID diagnosis were age-referenced and gathered only after the exclusion of clinical-laboratory signals of infections at the time of blood sampling at least 6 months from the last administration of steroids or other immunosuppressants and before starting chemoprophylaxis and IgRT (34–36).

### 2.4 Molecular analysis

A targeted gene panel including 46 genes causative for agammaglobulinemia, CVID, and IEIs with immune dysregulation was applied to 14/25 patients with CVID (56%), while a comparative genomic hybridization array was performed only in a single case (4%).

### 2.5 Statistical analysis

Descriptive statistics included the mean (95% confidence interval) and the frequency for continuous and categorical variables, respectively. Analyses were performed using STATA software version 7.0 and Microsoft Excel version 2013.

The data analysis aimed to assess the statistical significance (two-tailed p-value of <0.05) of differences through chi-squared tests for frequency and Student’s t-test for the mean, and to describe the correlation (two-tailed p-value of <0.05) between variables through the Pearson correlation coefficient.

## 3 Results

### 3.1 Clinical characterization and comparison between GLILD+ and GLILD- patients

In total, 25 patients with a diagnosis of CVID who received an HRCT were included in the study. In 9, a diagnosis of GLILD was confirmed by HRCT, while 16 were included as the control group. The clinical characteristics of the study cohort are summarized in **Table 1**.

**Table 1:** Clinical characteristics of patients with and without GLILD.

Variables	GLILD+ group (n=9)	GLILD- group (n=16)
Age at CVID diagnosis, year, median (range)	41 (10-60)	38 (13-60)
Sex, M/F, n	2/7	7/9
Infections, n (%)	7 (78)	12 (75)
Respiratory findings (except for GLILD), n (%)	3 (33)	6 (38)
Gastrointestinal findings, n (%)	4 (44)	9 (56)
Autoimmune manifestations, n (%)	4 (44)	6 (38)
Lymphoproliferative manifestations, n (%)	9 (100)	10 (63)

CVID, common variable immunodeficiency; F, female; GLILD, granulomatous lymphocytic interstitial lung disease; M, male.

Median age at CVID diagnosis was 41 years (range, 10–60) for the GLILD group and 38 years (range, 13–60) for the control group. A similar percentage of patients in both groups presented with infections as part of the clinical spectrum of the disease (78% vs. 75%).

Excluding the GLILD phenotype, nine patients among both groups presented with additional pulmonary findings, including bronchiectasis in five, sarcoidosis in one, pulmonary nodules in one, interstitial lung disease in one, and CMV pneumonia in two patients.

Gastrointestinal manifestations were present in 13/25 patients, including IBD-like colitis in seven, celiac disease in three, and non-specific gastrointestinal symptoms of unknown origin in three patients.

Autoimmune comorbidities were present in 10 patients, including autoimmune thrombocytopenia in six, celiac disease in three, autoimmune thyroiditis in four, and autoimmune hepatitis in one.

Lymphoproliferative findings, detected in 19 patients, included lymphomas in 5, hepato-splenomegaly in 11, and non-malignant lymphoproliferation in 8 patients. As concerns a definite molecular diagnosis, it was achieved in 1/9 GLILD+ and 2/16 GLILD- patients, as extensively reported in **Table 2**.

**Table 2:** Molecular characteristics of patients with and without GLILD.

Variables	GLILD+ group (n=9)	GLILD-group (n=16)
IEI gene panel, n (%)	5 (55)	8 (50)
ALPS gene panel, n (%)	1 (11)	0 (0)
CGH array, n (%)	1 (11)	0 (0)
Molecular IEI diagnosis, n (%)	1 (11) • *CR2*:c.457G>T_p.Glu153Ter heterozygous pathogenic variant	2 (12) • *TNFRSF13B*:c.310T>C_p.Cys104Arg heterozygous variant with conflicting classifications of pathogenicity • *PRF1*:c.272C>T_p.Ala91Val heterozygous variant with conflicting classifications of pathogenicity • *CECR1*:c.144delG_p.Arg49Glyfs and *CECR1*:c.1085G>A_p.Trp362Ter compound heterozygous likely pathogenic variants leading to ADA2 deficiency diagnosis • *NFKB1*:c.2804G>A_p.Arg935His heterozygous variant of uncertain significance • *AICDA*:c.361G>C heterozygous variant of uncertain significance • *NFKB1*:c.469C>T_p.Arg157Ter heterozygous pathogenic variant leading to genetic CVID diagnosis • *TNFRSF13B*:c.310T>C_p.Cys104Arg heterozygous variant with conflicting classifications of pathogenicity and *IGLL1*:c.350C>T_p.Thr117Ile heterozygous variant with conflicting classifications of pathogenicity • *ATM*:c.5262G>T_p.Lys1754Asn heterozygous variant of uncertain significance

ALPS, autoimmune lymphoproliferative syndrome; CGH, comparative genomic hybridization; CVID, common variable immunodeficiency; GLILD, granulomatous lymphocytic interstitial lung disease; IEI; Inborn Errors of Immunity.

### 3.2 Immunophenotype comparison in the GLILD+ and GLILD- groups

Immunophenotyping revealed an absolute leukocyte and lymphocyte and relative CD3+ PAN-T cell increase; an absolute CD4+ and CD8+ T-cell expansion skewed towards a prevalence of the effector subsets in face of a relative naïve and regulatory T-cell (T-reg) diminishment; specular CD19+ naïve, transitional, and CD21low B subsets predominance on memory; and switched memory B lymphocytes in the GLILD+ group compared to the controls.

As regards other subsets, the absolute natural killer (NK) cell mean count was higher in the GLILD+ group, while the relative TCRαβ+CD3+CD4-CD8- double negative T (DNT) and CD3+γ+δ+ cell mean counts were lower.

As shown in **Tables 3** and **4**, there were statistically significant differences between the two groups for the mean relative count of Treg cells, CD8+ naïve and central memory T-cells, DNT cells, PAN-B, naïve, memory, switched memory, and CD21low B-cells.

**Table 3:** Extended immuno-hematological comparison between the GLILD+ and GLILD- groups (quantitative variables) at timepoints T0 (at CVID diagnosis) and T1 (on IRT therapy) (SD, standard deviation).

Variables		Time point	GLILD+ group (n=8)		GLILD- group (n=17)		T-test
			Obs	Mean [SD]	Obs	Mean [SD]	P-value
*Basic features*	WBC (cell/ul)	T0	7	6 774.3 [2 106.1]	12	5 971.7 [2 500.5]	0.253
		T1	9	6 942.2 [5 068.4]	13	5 520.8 [2 875.1]	0.462
	Hemoglobin (g/dl)	T0	7	13.9 [1.9]	12	14.8 [1.7]	0.32
		T1	9	13.8 [1.1]	13	13.8 [1.6]	1
	Platelets (cellx10³/ul)	T0	7	219.0 [108.4]	12	191.5 [101.5]	0.595
		T1	9	174.7 [83.2]	13	222.8 [95.6]	0.225
	Neutrophils (cell/ul)	T0	7	3 481.4 [937.5]	12	3 689.2 [1 556.5]	0.719
		T1	9	3 707.8 [2 352.3]	13	3 341.5 [1 921.0]	0.702
	Eosinophils (cell/ul)	T0	7	192.8 [220.6]	12	283.3 [503.0]	0.596
		T1	9	126.7 [86.4]	13	136.1 [151.2]	0.777
	Lymphocytes (cell/ul)	T0	7	2 618.6 [1 462.2]	12	1 776.7 [605.3]	0.189
		T1	9	2 546.7 [2 739.3]	13	1 656.9 [961.4]	0.373
	CD3+ PAN-T cells (%†)	T0	7	76.0 [6.8]	12	72.5 [11.0]	0.404
		T1	9	80.4 [8.8]	13	77.8 [11.4]	0.543
	CD4+/CD8+ ratio	T0	7	1.5 [0.8]	12	1.4 [0.4]	0.952
		T1	9	1.2 [0.8]	13	1.2 [0.4]	0.865
*CD4+ T-cell subsets*	CD3+CD4+ T cells (cell/ul)	T0	7	789.7 [512.1]	12	728.3 [247.8]	0.774
		T1	9	943.9 [889.6]	13	613.7 [262.9]	0.308
	CD3+CD4+ T cells (%†)	T0	7	41.3 [9.1]	12	40.7 [9.8]	0.781
		T1	9	38.3 [12.8]	13	40.2 [9.1]	0.891
	CD4+CD45RA+CCR7+ naїve T cells (%‡)	T0	6	22.3 [19.2]	12	28.0 [16.2]	0.551
		T1	9	14.1 [8.4]	12	24.1 [14.7]	0.063
	CD4+CD45RA-CCR7+ central memory T cells (%‡)	T0	6	51.8 [14.9]	12	47.6 [20.6]	0.625
		T1	9	48.7 [18.0]	12	48.4 [13.6]	0.966
	CD4+CD45RA-CCR7- effector memory T cells (%‡)	T0	9	29.8 [14.4]	12	20.7 [14.2]	0.231
		T1	8	24.5 [16.9]	12	22.7 [15.7]	0.812
	CD4+CD45RA+CCR7- terminal effector memory T cells (%‡)	T0	6	4.7 [5.1]	12	5.1 [10.1]	0.911
		T1	9	11.2 [20.2]	12	4.6 [11.5]	0.395
	CD4+CD127-CCR7+CD25++ regulatory T cells (%‡)	T0	6	2.2 [0.7]	12	3.3 [1.5]	*0.044
		T1	9	1.7 [1.2]	12	3.0 [2.2]	0.091
*CD8+ T-cell subsets*	CD3+CD8+ T cells (cell/ul)	T0	7	868.7 [699.5]	12	504.6 [180.6]	0.222
		T1	9	1 210.1 [1 622.4]	13	584.7 [406.2]	0.288
	CD3+CD8+ T cells (%†)	T0	7	32.7 [10.9]	12	28.7 [5.7]	0.398
		T1	9	38.8 [16.8]	13	34.8 [7.5]	0.517
	CD8+CD45RA+CCR7+ naїve T cells (%§)	T0	6	12.3 [8.1]	12	25.0 [13.5]	*0.025
		T1	9	9.6 [9.2]	12	24.5 [19.8]	*0.035
	CD8+CD45RA-CCR7+ central memory T cells (%§)	T0	6	7.6 [7.4]	12	11.3 [5.4]	0.314
		T1	9	4.5 [4.5]	12	11.2 [8.5]	*0.033
	CD8+CD45RA-CCR7- effector memory T cells (%§)	T0	6	34.8 [19.3]	12	29.8 [14.6]	0.587
		T1	9	33.9 [20.7]	12	25.7 [11.2]	0.308
	CD8+CD45RA+CCR7- late effector T cells (%§)	T0	6	45.2 [23.6]	12	31.8 [18.7]	0.261
		T1	9	52.1 [21.2]	12	37.1 [19.5]	0.115
*Other cell subsets*	CD56+CD16+CD3- natural killer cells (cell/ul)	T0	7	282.0 [227.5]	12	211.0 [159.5]	0.484
		T1	9	321.4 [517.8]	13	179.6 [174.9]	0.449
	CD56+CD16+CD3- natural killer cells (%†)	T0	7	11.6 [5.5]	12	11.8 [6.2]	0.926
		T1	9	12.7 [10.6]	13	10.4 [5.6]	0.566
	TCRαβ+CD3+CD4-CD8- double negative T cells (%¶)	T0	6	1.4 [0.5]	12	1.8 [1.2]	0.29
		T1	9	0.7 [0.7]	12	2.0 [1.6]	*0.035
	CD3+γ+δ+ (%†)	T0	7	2.4 [1.6]	12	3.7 [2.7]	0.225
		T1	9	2.7 [1.6]	12	3.7 [2.5]	0.277
*CD19+ B-cell subsets*	CD19+ PAN-B cells (cell/ul)	T0	7	130.0 [94.1]	12	258.3 [191.9]	0.068
		T1	9	183.3 [296.9]	13	218.1 [231.6]	0.772
	CD19+ PAN-B cells (%†)	T0	7	6.5 [4.7]	12	14.7 [8.5]	*0.015
		T1	9	6.1 [4.3]	13	10.8 [9.4]	0.127
	CD19+IgD+CD27- naïve B cells (%††)	T0	6	83.0 [13.3]	12	64.6 [22.7]	*0.047
		T1	8	83.1 [11.6]	11	65.9 [27.0]	0.079
	CD19+IgM++CD38++ transitional B cells (%††)	T0	6	4.6 [3.2]	11	3.7 [3.7]	0.593
		T1	8	4.0 [6.7]	10	1.5 [1.6]	0.334
	CD19+IgD+CD27+ memory B cells (%††)	T0	6	7.7 [9.6]	12	23.8 [18.2]	*0.026
		T1	8	22.5 [24.8]	10	37.0 [33.6]	0.31
	CD19+IgD-CD27+ switched memory B cells (%††)	T0	6	0.6 [0.7]	12	4.3 [4.4]	*0.014
		T1	8	0.3 [0.7]	10	2.7 [2.7]	*0.024
	CD19+CD21+lCD38- CD21low B cells (%††)	T0	6	28.7 [15.0]	11	9.4 [5.8]	*0.024
		T1	9	14.1 [6.7]	11	14.7 [13.1]	0.89
	CD19+IgM-+CD38++ plasmablasts (%††)	T0	6	0.1 [0.1]	11	0.2 [0.6]	0.454
		T1	8	0.0 [0.1]	10	0.1 [0.1]	0.523
*Immunoglobulin levels*	IgG (mg/dl)‡‡	T0	9	413.0 [373.8]	12	611.8 [208.8]	0.177
		T1	9	832.2 [248.7]	13	904.4 [242.9]	0.508
	IgA (mg/dl)‡‡	T0	9	7.9 [10.3]	12	33.1 [32.3]	*0.024
		T1	9	7.3 [9.4]	13	21.5 [29.8]	0.129
	IgM (mg/dl)‡‡	T0	9	36.4 [31.0]	12	65.8 [62.9]	0.178
		T1	9	63.0 [111.3]	13	35.8 [47.5]	0.505
	IgE (IU/ml)	T0	6	0.7 [1.6]	12	86.4 [232.3]	0.227
		T1	9	0.4 [1.3]	13	9.2 [22.6]	0.187
	IgG1 (mg/dl)‡‡	T0	5	201.8 [215.2]	9	285.8 [77.5]	0.441
		T1	5	409.6 [67.8]	7	486.0 [194.9]	0.366
	IgG2 (mg/dl)‡‡	T0	5	70.1 [100.3]	9	188.4 [89.4]	0.061
		T1	5	287.1 [23.8]	7	391.1 [165.8]	0.15
	IgG3 (mg/dl)‡‡	T0	5	21.8 [20.2]	9	44.6 [29.1]	0.113
		T1	5	33.5 [22.6]	7	46.5 [26.9]	0.387
	IgG4 (mg/dl)‡‡	T0	5	2.6 [2.0]	9	12.3 [12.7]	*0.030
		T1	5	13.8 [5.4]	7	14.4 [10.4]	0.903
*Complement levels*	C3 (mg/dl)	T0	4	133.2 [25.4]	12	126.1 [18.5]	0.63
		T1	9	145.4 [27.5]	12	133.6 [27.0]	0.359
	C4 (mg/dl)	T0	4	33.7 [8.2]	12	36.7 [14.5]	0.62
		T1	9	33.5 [13.1]	12	43.2 [20.1]	0.201

CVID, common variable immunodeficiency; GLILD, granulomatous lymphocytic interstitial lung disease; IRT, immunoglobulin replacement therapy; obs, observations; WBC, white blood cells.

* Statistically significant.

† % total lymphocytes.

‡ % total CD4+ cells.

§ % total CD8+ cells.

¶ % TCRαβ+CD3+ cells.

†† % total CD19+ cells.

‡‡ SI conversion factor: To convert IgG/IgA/IgM to g/L, multiply values by 10².

**Table 4:** Extended immuno-hematological comparison between the GLILD+ and GLILD- groups (qualitative variables) at timepoints T0 (at CVID diagnosis) and T1 (on IRT therapy) (SD: standard deviation).

Variables		Time point	GLILD+ group (n=8)		GLILD- group (n=17)		Fisher
			Obs	Prevalence (%)	Obs	Prevalence (%)	P-value
*Basic features*	↓ WBC (cell/ul)	T0	7	0 (0)	12	0 (0)	1.000
		T1	9	1 (11)	13	2 (15)	1.000
	↑ Hemoglobin (g/dl)	T0	7	1 (14)	12	4 (33)	0.603
		T1	9	0 (0)	13	3 (23)	0.240
	↓ Platelets (cellx10³/ul)	T0	7	2 (29)	12	5 (42)	0.656
		T1	9	5 (55)	13	3 (23)	0.187
	↓ Neutrophils (cell/ul)	T0	7	0 (0)	12	1 (8)	1.000
		T1	9	0 (0)	13	2 (15)	0.493
	↑ Eosinophils (cell/ul)	T0	7	1 (14)	12	2 (17)	1.000
		T1	9	0 (0)	13	1 (8)	1.000
	↓ Lymphocytes (cell/ul)	T0	7	2 (29)	12	0 (0)	1.000
		T1	9	3 (33)	13	5 (38)	1.000
	↑ CD3+ PAN-T cells (%†)	T0	7	0 (0)	12	0 (0)	1.000
		T1	9	1 (11)	13	1 (8)	1.000
	↓ CD4+/CD8+ ratio	T0	7	1 (14)	12	1 (8)	1.000
		T1	9	4 (44)	13	4 (31)	0.662
*CD4+ T-cell subsets*	↓ CD3+CD4+ T cells (cell/ul)	T0	7	1 (14)	12	2 (17)	1.000
		T1	9	4 (44)	13	5 (38)	1.000
	↓ CD3+CD4+ T cells (%†)	T0	7	0 (0)	12	1 (8)	1.000
		T1	9	1 (11)	13	2 (15)	1.000
	↓ CD4+CD45RA+CCR7+ naїve T cells (%‡)	T0	6	2 (33)	12	4 (33)	1.000
		T1	9	5 (55)	12	4 (33)	0.396
	↓ CD4+CD45RA-CCR7+ central memory T cells (%‡)	T0	6	0 (0)	12	0 (0)	1.000
		T1	9	1 (11)	12	1 (8)	1.000
	↑ CD4+CD45RA-CCR7- effector memory T cells (%‡)	T0	6	4 (67)	12	3 (25)	0.141
		T1	9	5 (55)	12	4 (33)	0.396
	↑ CD4+CD45RA+CCR7- terminal effector memory T cells (%‡)	T0	6	2 (33)	12	2 (17)	0.569
		T1	9	3 (33)	12	1 (8)	0.272
	↓ CD4+CD127-CCR7+CD25++ regulatory T cells (%‡)	T0	6	6 (100)	12	7 (58)	0.114
		T1	9	8 (89)	12	7 (58)	0.178
*CD8+ T-cell subsets*	↓ CD3+CD8+ T cells (cell/ul)	T0	7	0 (0)	12	1 (8)	1.000
		T1	9	0 (0)	13	2 (15)	0.493
	↑ CD3+CD8+ T cells (%†)	T0	7	1 (14)	12	1 (8)	1.000
		T1	9	2 (22)	13	4 (31)	1.000
	↓ CD8+CD45RA+CCR7+ naїve T cells (%§)	T0	6	2 (33)	12	0 (0)	0.098
		T1	9	4 (44)	12	1 (8)	0.119
	↑ CD8+CD45RA-CCR7+ central memory T cells (%§)	T0	6	0 (0)	12	1 (8)	1.000
		T1	9	0 (0)	12	2 (17)	0.486
	↓ CD8+CD45RA-CCR7- effector memory T cells (%§)	T0	6	2 (33)	12	1 (8)	0.245
		T1	9	2 (22)	12	2 (17)	1.000
	↑ CD8+CD45RA+CCR7- late effector T cells (%§)	T0	6	2 (33)	12	2 (17)	0.569
		T1	9	3 (33)	12	2 (17)	0.611
*Other cell subsets*	↓ CD56+CD16+CD3- natural killer cells (cell/ul)	T0	7	0 (0)	12	3 (25)	0.263
		T1	9	3 (33)	13	5 (38)	1.000
	↓ CD56+CD16+CD3- natural killer cells (%†)	T0	7	1 (14)	12	2 (17)	1.000
		T1	9	2 (22)	13	1 (8)	0.544
	↓ TCRαβ+CD3+CD4-CD8- double negative T cells (%¶)	T0	6	0 (0)	12	0 (0)	1.000
		T1	9	3 (33)	12	1 (8)	0.272
	↓ CD3+γ+δ+ (%†)	T0	7	0 (0)	12	0 (0)	1.000
		T1	9	1 (11)	12	0 (0)	0.429
*CD19+ B-cell subsets*	↑ CD19+ PAN-B cells (cell/ul)	T0	7	1 (14)	12	4 (33)	0.603
		T1	9	1 (11)	13	4 (31)	0.360
	↑ CD19+ PAN-B cells (%†)	T0	7	1 (14)	12	7 (58)	0.147
		T1	9	1 (11)	13	4 (31)	0.360
	↓ CD19+IgD+CD27- naïve B cells (%††)	T0	6	0 (0)	12	5 (42)	0.114
		T1	8	0 (0)	11	4 (36)	0.103
	↓ CD19+IgM++CD38++ transitional B cells (%††)	T0	6	1 (17)	11	3 (27)	1.000
		T1	8	2 (25)	10	6 (60)	0.188
	↑ CD19+IgD+CD27+ memory B cells (%††)	T0	6	1 (17)	12	6 (50)	0.316
		T1	8	3 (27)	10	5 (50)	0.664
	↓ CD19+IgD-CD27+ switched memory B cells (%††)	T0	6	6 (100)	12	10 (83)	0.530
		T1	8	8 (100)	10	10 (100)	1.000
	↑ CD19+CD21+lCD38- CD21low B cells (%††)	T0	6	6 (100)	11	8 (73)	0.515
		T1	9	8 (89)	11	9 (82)	1.000
	↓ CD19+IgM-+CD38++ plasmablasts (%††)	T0	6	5 (83)	11	2 (18)	0.068
		T1	8	8 (100)	10	10 (100)	1.000
*Immunoglobulin levels*	↓ IgG (mg/dl)‡‡	T0	9	7 (78)	12	10 (83)	1.000
		T1	9	4 (44)	13	5 (38)	1.000
	↓ IgA (mg/dl)‡‡	T0	9	9 (100)	12	7 (58)	*0.045
		T1	9	9 (100)	13	11 (85)	0.493
	↓ IgM (mg/dl)‡‡	T0	9	8 (89)	12	8 (67)	0.338
		T1	9	7 (78)	13	11 (85)	1.000
	↑ IgE (IU/ml)	T0	6	0 (0)	12	2 (17)	0.529
		T1	9	0 (0)	13	0 (0)	1.000
	↓ IgG1 (mg/dl)‡‡	T0	5	4 (80)	9	9 (100)	0.357
		T1	5	5 (100)	7	4 (57)	0.204
	↓ IgG2 (mg/dl)‡‡	T0	5	4 (80)	9	4 (44)	0.301
		T1	5	0 (0)	7	0 (0)	1.000
	↓ IgG3 (mg/dl)‡‡	T0	5	3 (60)	9	1 (11)	0.095
		T1	5	1 (20)	7	1 (14)	1.000
	↓ IgG4 (mg/dl)‡‡	T0	5	5 (100)	9	6 (67)	0.258
		T1	5	2 (40)	7	3 (43)	1.000
*Autoimmunity and thyroid function*	↑ ANA (at least 1:80 titre)	T0	6	2 (33)	13	2 (15)	0.557
		T1	9	1 (11)	11	1 (9)	1.000
	↑ Anti-TG antibodies (> 4 IU/ml)	T0	5	1 (20)	12	3 (25)	1.000
		T1	6	2 (33)	7	3 (43)	1.000
	↑ Anti-TPO antibodies (> 9 IU/ml)	T0	5	1 (20)	12	3 (25)	1.000
		T1	5	2 (40)	8	6 (75)	0.293
	↑ TSH (> 4.5 uU/ml)	T0	7	1 (14)	13	2 (15)	1.000
		T1	7	1 (14)	13	0 (0)	0.350
	↑ FT4 (> 12 pg/ml)	T0	3	1 (33)	5	1 (20)	1.000
		T1	5	0 (0)	1	0 (0)	1.000
	↑ Anti-gliadin antibodies (> 10 IU/ml)	T0	5	0 (0)	7	1 (14)	1.000
		T1	7	0 (0)	6	0 (0)	1.000

ANA, anti-nuclear antibodies; CVID, common variable immunodeficiency; GLILD, granulomatous lymphocytic interstitial lung disease; IRT, immunoglobulin replacement therapy; obs, observations; TG, thyroglobulin; TPO, thyroperoxidase; TSH, thyroid stimulating hormone; WBC, white blood cells.

Legend: ↓, decrease; ↑, increase.

* Statistically significant.

† % total lymphocytes.

‡ % total CD4+ cells.

§ % total CD8+ cells.

¶ % TCRαβ+CD3+ cells.

†† % total CD19+ cells.

‡‡ SI conversion factor: To convert IgG/IgA/IgM to g/L, multiply values by 10².

As concerns T-cell subsets, the mean T-reg relative count was inferior (p = 0.044) in the GLILD+ group, as was the mean CD8+ naïve (p = 0.025), central memory (p = 0.033), and DNT cell (p = 0.035) relative counts (**Table 3**).

Considering the B-cell compartment, the CD19+ PAN-B cell percentage was, on average, inferior (p = 0.015) in the GLILD+ group, and this was similar for memory (p = 0.026) and switched memory B-cell percentages (p = 0.014), while naïve (p = 0.047) and CD21low (p = 0.024) B-cell relative counts were, on average, superior in the GLILD+ group (**Table 3**).

### 3.3 Immunoglobulin concentrations

Immunoglobulin concentrations were detected at two different time points: at CVID diagnosis before the start of IgRT (T0), and while on substitutive therapy (T1).

As shown in **Table 3**, all immunoglobulin classes (IgG and subclasses, IgA, IgM, and IgE) were, on average, lower in the GLILD+ group at both time points, with IgA (p = 0.024) and IgG4 (p = 0.030) being significantly inferior in the GLILD+ group at CVID diagnosis.

Furthermore, an IgA reduction under the age- and sex-matched lower limit of normal was significantly more frequent (p = 0.045) in the GLILD+ group at CVID diagnosis (**Table 4**).

### 3.4 An unedited case series on GLILD management with a focus on acute SARS-CoV-2 and chronic CMV infection during immunosuppressive treatments

Given that GLILD management lacks consensus guidelines based on solid evidence and is primarily guided by expert opinions and internal protocols that vary across centers, there is a significant gap in the literature regarding the management of acute SARS-CoV-2 and chronic CMV infections in patients with CVID with GLILD undergoing immunosuppressive therapy. Here, we present a detailed analysis of our 9-patient GLILD cohort, comparing their pulmonary radiological and functional profiles before and after immunosuppressive treatment.

1) The first case involves a 22-year-old male who has been on IgRT since the age of 15 following a CVID diagnosis. His clinical history is characterized by early-onset ITP in infancy, later complicated during adolescence by hepatosplenomegaly and abdominal and thoracic polyclonal lymphoproliferation. GLILD was suspected after lung imaging was performed at age 17 for severe CMV pneumonia, which required treatment with ganciclovir and later valganciclovir until September 2019.

Following the discontinuation of antiviral therapy, thoracic HRCT revealed an increase in both the number and size of multiple parenchymal opacities. These included predominantly peripheral nodular lesions with a pleural base and consolidative opacities with air bronchograms in the right middle lobe and right postero-basal region. Additional lesions with ground-glass density were observed peripherally and at the hilum. Extensive mediastinal and hilar lymphadenopathy was present, affecting all lymph node stations, with the most prominent adenopathies located in the subcarinal region (5 cm×3 cm), anterior mediastinum (thymic area, 35 mm×18 mm), Barety’s loggia (27 mm×15 mm), and right hilum (30 mm×20 mm). LFTs identified a mild restrictive ventilatory defect. A transbronchial biopsy ruled out malignancy, leading to a GLILD diagnosis at 18 years of age.

Treatment with mycophenolate mofetil (MMF) at 2 g/day was initiated in August 2020, resulting in significant clinical, functional, and radiological improvement within 3 months. The patient became asymptomatic, with improvements in FEV1, FVC, and DLCO, along with a reduction in ground-glass opacities, parenchymal consolidations, and mediastinal lymph node size. Treatment was well-tolerated, despite a pauci-symptomatic SARS-CoV-2 infection in August 2021, which was successfully managed with casirivimab and imdevimab.

**Figure 1:**
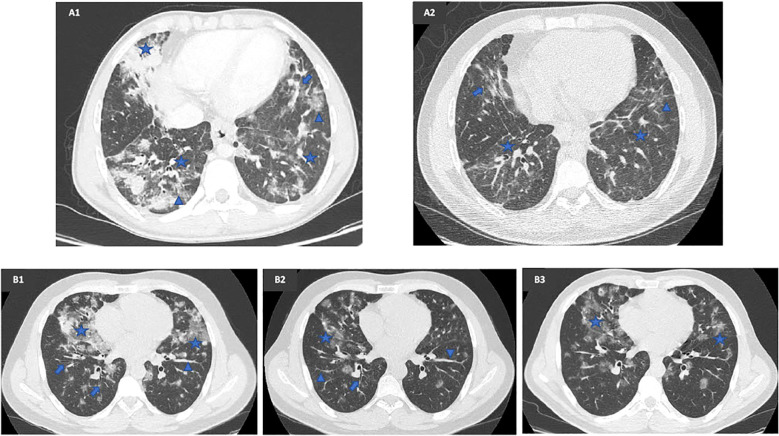
Radiological comparison before and after mycophenolate mofetil (MMF) therapy in two patients with a CVID-related GLILD complicated by a chronic CMV infection and SARS-CoV-2 infection. **(A1)** Lung HRCT executed in October 2019, once recovered from CMV pneumonia, prior to MMF therapy, showing bilateral mixed ground-glass (arrowhead) and solid nodules (star) along with inflammatory and fibrotic lines (arrow). **(A2)** Lung HRCT executed in April 2022, 20 months after MMF start, during CMV pneumonia, showing a sharp improvement in number, density, and size of bilateral parenchymal nodules. **(B1)** Baseline lung HRCT executed in November 2019, prior to MMF therapy, showing multiple diffuse bilateral parenchymal thickenings (arrowhead) with ground-glass density (star) and bronchiectasis (arrow), especially on the right side. **(B2)** Lung HRCT executed in February 2021, 7 months after MMF start, showing a considerable cleansing of the multiple ground-glass areas (star), with persistence of parenchymal thickening (arrowhead) and bronchiectasis (arrow). **(B3)** Lung HRCT executed in August 2022, 26 months after MMF start, showing disease progression with reappearance of ground-glass nodules (star) bilaterally.

A lung HRCT performed in February 2022 indicated disease progression, showing increased ground-glass opacities with a cotton-like, more consolidated appearance in the anterior segment of the left lower lobe and the apical-dorsal segment of the left upper lobe. Despite this, the patient maintained a favorable clinical and functional response to MMF. CMV blood viral load fluctuated between undetectable and 889 DNA copies/ml until April 2022, when he developed another episode of CMV pneumonia, necessitating MMF discontinuation. Notably, despite the pneumonia, an HRCT in April 2022 demonstrated a marked reduction in the number, density, and size of bilateral parenchymal nodules (**Figure 1A**).

The patient experienced additional clinically significant mixed viral-bacterial airway infections requiring antibiotic therapy in July and November 2022. HRCT at these time points revealed further parenchymal lesions indicative of GLILD relapse. In response, he was treated with two rituximab injections (1 g/dose) administered 2 weeks apart in April and May 2023; however, this approach was unsuccessful.

At the most recent evaluation in December 2023, DLCO remained slightly reduced, and HRCT showed new-onset bilateral diffuse ground-glass opacities. The clinical picture was dominated by recurrent febrile airway infections.

2) The second case involves a 36-year-old male who has been on IgRT since the age of 28 following a CVID diagnosis. The initial manifestation was mild thrombocytopenia in late puberty, followed by episodes of giardiasis and campylobacteriosis. CVID and GLILD were suspected concurrently after two episodes of severe, slow-resolving, antibiotic-resistant pneumonia. Once the patient fully recovered, imaging revealed multiple diffuse centrilobular parenchymal thickenings with ground-glass density, distributed bilaterally with a predominance in the middle lobe and lingular region, while sparing the apical regions. These findings, suggestive of alveolar proteinosis, were associated with hilar-mediastinal adenopathy and splenomegaly.

Over time, the patient developed recurrent respiratory symptoms and was diagnosed with steroid-dependent cryptogenic organizing pneumonia. A VATS biopsy at age 29 excluded malignancy, confirming the diagnosis. He was initially treated with prednisone at 50 mg/day starting in October 2017, which was gradually tapered and discontinued by September 2018. Subsequent courses of prednisone at 25 mg/day were administered and weaned off from February to May 2019 and again from November 2019 to June 2020.

To address steroid dependence, a multidisciplinary evaluation recommended MMF therapy at 2 g/day, which commenced in June 2020. After 7 months, the patient demonstrated a favorable clinical, functional, and radiological response, characterized by the absence of respiratory symptoms, normalization of FEV1, FVC, and DLCO, and a reduction in ground-glass opacities with partial resolution of previously observed parenchymal thickening (**Figure 1B**). MMF was well-tolerated, with CMV blood viral load ranging from <300 to 513 DNA copies/ml, and the patient experienced a pauci-symptomatic SARS-CoV-2 infection in April 2022.

A notable discrepancy emerged between clinical findings, LFTs, and radiological patterns in response to MMF. While the patient remained asymptomatic and maintained normal pulmonary function, thoracic HRCTs performed in December 2021 and August 2022 showed slow but continuous disease progression, with new consolidative changes and ground-glass opacities in the middle lobe (**Figure 1B**). Given this discordance, MMF was discontinued in October 2022, and the patient received two rituximab infusions (1 g/dose) administered 2 weeks apart in March and April 2023.

At the 3-month follow-up, a combined clinical and functional improvement was observed, which was radiologically confirmed 3 months later. The HRCT in October 2023 demonstrated a numerical and dimensional reduction in pseudonodular, subsolid-density, ground-glass-like lesions, which were bilaterally distributed in a peribronchovascular pattern, particularly in the upper and middle lung fields.

3) The third case involves a 70-year-old woman on IgRT since February 2021 for late-onset CVID. Her clinical history includes Hashimoto thyroiditis-related chronic giant urticaria diagnosed at age 27, followed by abdominal and thoracic polyclonal lymphoproliferation emerging in her 60s. She later developed severe refractory steroid-resistant ITP, necessitating rituximab and subsequent eltrombopag treatment starting in January 2021. Additionally, she was diagnosed with psoriasis in 2016 and underwent treatment with steroids for 6 months, methotrexate for 1 year, and ustekinumab for 2 years until August 2019.

GLILD was first suspected following a lung CT in December 2019, performed at age 65 due to persistent chronic cough, dyspnea, and fatigue. Imaging revealed multiple dense nodular areas surrounded by ground-glass opacities in the dorsal segments of the upper lobes and basal pyramids of the lower lobes. Additional findings included thickened interstitial septa at the lung bases and multiple mediastinal, axillary, and retroperitoneal adenopathies, suggestive of non-specific interstitial pneumonia with a possible ustekinumab-related lung injury.

The patient underwent a steroid regimen (50 mg/day), which was gradually tapered and discontinued between August and October 2020, resulting in an optimal clinical and radiological response. HRCT confirmed complete resolution of the ground-glass opacities, although interstitial septal thickening at the lung bases persisted. However, respiratory symptoms rapidly recurred after steroid discontinuation. A follow-up lung HRCT in February 2021 revealed pulmonary features consistent with organizing pneumonia, along with stable interstitial septal thickening in the basal regions and a slight enlargement of multiple thoracic lymph nodes.

**Figure 2:**
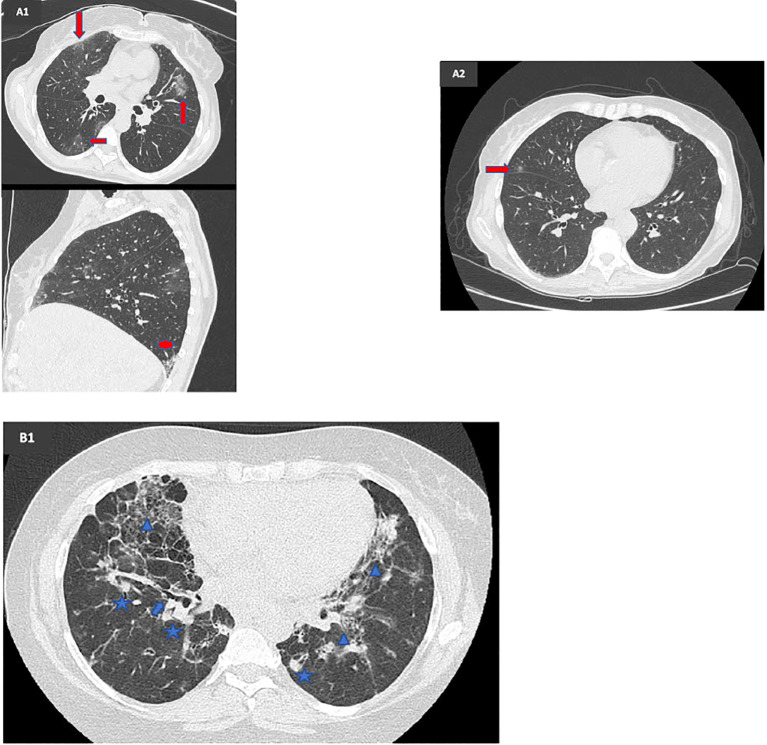
Radiological comparison before and after mycophenolate mofetil (MMF) therapy in two patients with a CVID-related GLILD complicated by SARS-CoV-2 infection **(A1)** Lung HRCT executed in February 2021, during a GLILD flare, prior to MMF therapy, showing multiple dense nodular areas (arrowhead) bilaterally with modest thickening of the interstitial septa (star) and ground-glass areas (arrow) especially in the left side. **(A2)** Lung HRCT executed in March 2022, 10 months after MMF start, showing a sharp radiological improvement with resolution of ground-glass areas. **(B1)** Lung HRCT executed in June 2020, prior to MMF therapy, showing multiple ground-glass (arrowhead) and dense nodular areas (star) distributed bilaterally, with interstitial reticular thickening and bronchiectasis foci (arrow).

In May 2021, MMF therapy was initiated, allowing for the gradual withdrawal of steroids. This led to a rapid clinical, functional, and radiological improvement, along with an associated stabilization in platelet counts, enabling the subsequent discontinuation of eltrombopag. The most recent HRCT in March 2022 confirmed complete resolution of the ground-glass opacities (**Figure 2A**), while pulmonary function tests conducted in September 2022 were within normal limits.

At the March 2023 follow-up, the patient remained clinically, functionally, and radiologically stable, except for the emergence of new parenchymal nodular opacities in the left lower lobe and lingula. These findings were fully resolved on HRCT in May 2023 after a 7-day course of amoxicillin-clavulanate and azithromycin.

MMF has been generally well-tolerated, despite a pauci-symptomatic SARS-CoV-2 infection in May 2022, which was successfully managed with protease inhibitors nirmatrelvir and ritonavir. However, the patient experienced non-specific ileocolitis with recurrent diarrhea and abdominal pain, which improved after reducing the MMF dosage from 2 g to 1 g/day in June 2023. This adjustment coincided with a progressive decline in platelet counts.

4) The fourth case describes a 44-year-old woman on IgRT since the age of 12, following a CVID diagnosis with early-onset manifestations, including persistent mild thrombocytopenia, recurrent respiratory and urinary tract infections, hepatosplenomegaly, and recurrent febrile granulomatous lymphadenitis-associated abdominal pain with giardiasis. In her late 20s, she was diagnosed with Hashimoto thyroiditis.

In 2017, she was hospitalized for a fever of unknown origin, later attributed to biopsy-confirmed immune-mediated hepatitis with superimposed CVID-related pre-sinusoidal portal hypertension, which was treated with steroids and taurocholic acid. During this hospitalization, GLILD was suspected due to lung HRCT findings, which revealed tralciform alveolar thickening and multiple bronchiectatic foci in the mid-basal regions of both lungs (right > left), along with enlarged hilar and mediastinal lymph nodes.

To manage both hepatic and pulmonary immune dysregulation, azathioprine was initiated, but it provided only a partial radiological response. The treatment continued until March 2018, when the patient developed sepsis-induced pancytopenia and severe CMV chorioretinitis, resulting in partial vision loss.

In January 2020, pulmonary function tests revealed a mild restrictive ventilatory defect with moderate DLCO reduction. Concurrently, lung HRCT showed multiple parenchymal consolidations and nodules bilaterally, with persistent interstitial reticular thickening and bronchiectatic foci predominantly in the lower lobes. These findings remained stable on follow-up imaging in June 2020. A transbronchial biopsy performed at age 41 confirmed GLILD, ruling out malignancy.

Given the functional and radiological pattern, MMF therapy (2 g/day) was initiated in April 2021. The patient exhibited a favorable functional and radiological response, with improvement in the nodular pattern as documented on HRCT in April 2022 (**Figure 2B**). Additionally, MMF contributed to a reduction in hepatotoxic and cholestatic indices, indicating a beneficial effect on liver immune dysregulation.

MMF was well-tolerated, with sustained suppression of CMV blood viral load. However, in August 2022, she experienced a symptomatic flu-like SARS-CoV-2 infection complicated by a secondary bacterial respiratory infection, successfully treated with nirmatrelvir/ritonavir, amoxicillin-clavulanate, and steroids. This episode was likely responsible for a slight worsening in pulmonary function tests at the last check-up in October 2022.

A combined functional and radiological evaluation in February 2023 showed stable DLCO and a bilateral reduction in the number and size of nodular and consolidative areas, although the fibrotic pattern remained unchanged. As a result, MMF was discontinued in May 2023 after 2 years of therapy.

The most recent evaluation in December 2023 confirmed sustained radiological stability despite a moderate DLCO decline (56%) and detectable Velcro-like crackles, particularly in the right basal lung field. These findings were likely associated with another flu-like SARS-CoV-2 infection in August 2023, which was treated with trimethoprim/sulfamethoxazole and nirmatrelvir/ritonavir.

5) The fifth case describes a 57-year-old woman with early-onset CVID, initially presenting with severe multi-refractory Evans syndrome, characterized by steroid-dependent AIHA and ITP resistant to multiple treatments. She underwent splenectomy at age 21, autologous hematopoietic stem cell transplantation following aggressive chemotherapy at age 42, and rituximab therapy from ages 43 to 47. Humoral immunodeficiency became evident at age 47, leading to the initiation of IgRT.

A diagnosis of Hyper-IgM-like CVID was established at age 54, prompted by a history of three pneumonia episodes requiring hospitalization in the preceding year, alongside a complex clinical-immunological-radiological profile marked by fungal susceptibility (manifesting as facial demodicosis), persistent CMV blood viral load <300 DNA copies/ml, and immune dysregulation, including:

- Aberrant malignant and non-malignant proliferation, including melanoma (age 33), abdominal and thoracic polyclonal lymphoproliferation, breast cancer (age 54), and medullary polyclonal expansion of large granular lymphocytes (age 56).
- Immune-mediated manifestations, such as acute medullary myelitis (age 40), hepatic fibrosis (age 56), and recurrent Evans syndrome relapses (ages 55, 56, and 57).
- GLILD, diagnosed at age 55 based on HRCT findings from June 2021 and February 2023, which demonstrated a progressive, widespread increase in small, blurred ground-glass areas, with the emergence of subcentimetric and centimetric nodular thickenings in the lower lung lobes bilaterally, along with interstitial disease features and early bronchiolectasis.

To manage GLILD, MMF therapy (2 g/day) was initiated in February 2023 but was provisionally interrupted in March 2023 and permanently discontinued in May 2023 due to gastrointestinal intolerance, including diarrhea and abdominal pain. Consequently, the patient received a four-dose rituximab cycle (375 mg/m² per dose) from May to June 2023. At the 6-month follow-up in December 2023, she demonstrated both functional and radiological improvement, despite persistent exertional dyspnea. HRCT findings showed resolution of most nodular thickenings in the lower lung lobes bilaterally, with only a residual 6 mm ground-glass area in the dorsal segment of the upper right lobe.

A 46-gene IEI panel was performed, revealing a *CR2*: c.457G>T (p.Glu153Ter) heterozygous pathogenic variant causative for CVID.

6) The sixth case involves a 57-year-old woman who has been on IgRT since age 53 following a CVID diagnosis with early-onset manifestations in infancy. Her clinical history includes recurrent upper and lower respiratory tract infections, which necessitated a tonsillectomy at age 6, and later decreased in frequency and severity with IgRT. Additional findings included ulcerative rectocolitis managed with mesalazine (diagnosed at age 46), pan-hypogammaglobulinemia (age 50), and splenomegaly (age 53).

In December 2021, she experienced a self-resolving, pauci-symptomatic SARS-CoV-2 infection, while chronic CMV infection has remained well-controlled, with viral loads consistently below 600 DNA copies/ml.

GLILD suspicion arose in 2019 following recurrent pneumonia episodes. Serial HRCT scans documented a progressive, widespread increase in the interlobular interstitial thickening, particularly in the lower lung lobes, along with transient and migratory nodular and pseudonodular thickenings in both peribronchial and peripheral regions. A transbronchial biopsy in 2022, at age 55, excluded malignancies and infections, confirming GLILD. To date, the patient has only shown mild functional impairment, not warranting specific treatment.

7) The seventh case concerns a woman whose CVID onset occurred in her early 30s, presenting with recurrent pneumonias requiring hospitalization, multiple HPV-related ano-genital condylomas, Bowenoid papulosis, molluscum contagiosum, annual orolabial HSV relapses, and two episodes of herpes zoster reactivation. Humoral immunodeficiency, manifesting as pan-hypogammaglobulinemia, became evident at age 37. CVID diagnosis was confirmed at age 39, following the previous year’s detection of splenic B-marginal zone lymphoma, which led to splenectomy.

Following CVID diagnosis, her clinical course was marked by a complex spectrum of infectious susceptibility and immune dysregulation, including:

- Immune-mediated disorders, such as sprue-like celiac disease (age 42) and a 3-year course of ITP (age 43).
- Severe pneumonia and chronic respiratory insufficiency, with recurrent infections due to *P. jirovecii* (2015), CMV (2015, 2019, 2021), and *L. pneumoniae* (2020), as well as JC virus-related progressive multifocal leukoencephalopathy (March 2022) and recurrent Tinea corporis affecting the lower limbs.
- Chronic CMV viremia, with peak viral loads reaching 9,728 DNA copies/ml, requiring prophylactic valganciclovir therapy.
- GLILD, first documented at age 43 via serial HRCTs, which showed progressive bronchial wall thickening, predominantly in the lower lung fields, along with an increasing number and size of parenchymal micronodules with a centrilobular and perilymphatic distribution and a ground-glass pattern, especially in the middle and lower lung fields.

Immunosuppressive treatment with prednisone (6.25 mg/day) was initiated in 2015 alongside trimethoprim/sulfamethoxazole chemoprophylaxis and nocturnal oxygen supplementation. Despite maintaining stable functional and radiological findings, the patient experienced progressive clinical deterioration, necessitating continuous oxygen therapy. In September 2022, she developed a mixed viral-bacterial pneumonia, ultimately leading to her passing at age 53.

8) The eighth case describes a 57-year-old woman who has been on IgRT since age 49 following a CVID diagnosis with late-onset manifestations in her 40s, characterized by recurrent upper and lower respiratory tract infections in the context of pan-hypogammaglobulinemia.

HRCT scans, starting in 2011, revealed bilateral peribronchial and subpleural pulmonary thickening with a relapsing and migratory pattern, displaying tree-in-bud and ground-glass features. Progressive hilar-mediastinal and axillary lymph node hyperplasia raised suspicion of GLILD, which was confirmed by transbronchial biopsy in February 2019 at age 52. Despite these findings, follow-up evaluations detected no significant functional impairment except for a slight decline in DLCO.

Her immune dysregulation spectrum also included:

- Nasal basal cell carcinoma in her 50s.
- Herpes zoster reactivation and polyclonal lymphoproliferation, associated with hepatosplenomegaly and thoracic/mesenteric lymphoid hyperplasia.

She experienced two self-resolving, pauci-symptomatic SARS-CoV-2 infections (July 2022 and August 2023). Future clinical, functional, and radiological follow-ups will determine whether rituximab therapy is warranted.

9) The ninth case involves a 37-year-old woman on IgRT since age 33, following an early-onset CVID diagnosis in infancy. Her initial clinical presentation included idiopathic chronic urticaria and recurrent respiratory and urinary tract infections, which decreased in frequency and severity with IgRT. Pan-hypogammaglobulinemia and hepatosplenomegaly were detected at ages 24 and 33, respectively.

She experienced two flu-like SARS-CoV-2 infections (November 2020 and August 2022); the former was treated with oral steroids and azithromycin, while the latter resolved spontaneously.

GLILD suspicion, confirmed by transbronchial biopsy in November 2021 (age 35), emerged in August 2019, coinciding with CVID diagnosis. Radiological findings showed a progressive appearance of multiple solid micro- and macro-nodules in all lung lobes. Some nodules exhibited small aerated bronchi, while others had a perilesional ground-glass halo, accompanied by diffuse peribronchial interstitial thickening and ubiquitous ground-glass nodulations.

Follow-up evaluations documented a gradual and persistent decline in DLCO, from 88% in 2019 to 60% in December 2023, alongside ongoing exertional dyspnea. Given this trend, a multidisciplinary discussion is planned to assess the potential initiation of rituximab therapy.

## 4 Discussion

To date, a comprehensive immunophenotypic characterization of patients with CVID-related GLILD, encompassing both T-cell and B-cell compartments, remains lacking in the literature. Our study contributes to bridging this gap by providing an in-depth analysis of the immunological profile of patients with GLILD. We systematically examined distinct T- and B-cell subsets while simultaneously conducting thorough clinical, pulmonary functional, and radiological assessments of our cohort. A similar comparative study between GLILD+ and GLILD- patients with CVID was recently performed by Cinetto et al (29).

### 4.1 Key strengths and contributions

One of the key strengths of our study is the extensive immunological analysis of CVID-related GLILD, encompassing nearly all T- and B-cell subsets. By correlating these immunological parameters with GLILD development and progression—both at CVID diagnosis and after the initiation of IgRT—we provide valuable clinical insights. Furthermore, our study offers a unique perspective on GLILD treatment, particularly in the context of chronic CMV and SARS-CoV-2 infections, by analyzing the clinical, functional, and radiological course of the disease throughout immunosuppressive therapy.

By performing systematic immunophenotypic comparisons at two different time points (T0 and T1), we demonstrated that statistically significant immunological differences between the GLILD+ and GLILD- groups are largely unaffected by IgRT. However, we observed notable shifts in certain parameters, with some previously significant differences becoming non-significant and *vice versa*. These findings suggest either an immunomodulatory effect of IgRT or persistent disease progression unresponsive to GLILD treatments.

### 4.2 T-cell subset differences between the GLILD+ and GLILD- groups

Our analysis of T-cell phenotypes revealed that the GLILD+ patients exhibited significantly lower mean relative count of DNT, CD4+ T-reg, CD8+ naive, and central memory T-cells compared to the controls. Additionally, although not statistically significant, there was a more pronounced predominance of CD4+ and CD8+ effector subsets over naive subsets. Given the lack of prior studies specifically comparing T-cell subsets between GLILD+ and GLILD- patients with CVID, these findings represent a novel contribution to the field.

We hypothesize that these differences may be attributed to chronic inflammation-driven immune activation, characteristic of GLILD-related polyclonal lymphoproliferation. The reduced number of Treg cells may fail to control this immune dysregulation, leading to an immunosenescent-like phenotype. This hypothesis aligns with findings from Fraz et al., who reported increased T-cell activation and exhaustion markers [mucin domain-containing protein 3 (TIM-3) and soluble IL-2Rα chain (CD25)] in patients with GLILD (37).

Bateman et al. previously associated T-cell abnormalities with polyclonal lymphoproliferation in CVID, demonstrating a significant reduction in total CD4+, CD4+ naive, and early differentiated CD4+ and CD8+ T-cells, along with an increase in CD8+ terminal effector memory T-cells. If non-malignant lymphoproliferation serves as a surrogate for GLILD, our results are consistent with these findings (38).

Interestingly, we observed an increased total lymphocyte count and elevated absolute and relative CD4+ and CD8+ T-cell counts in GLILD+ patients—findings that contradict results from Cinetto et al. (29), Bateman et al. (38), and Kellner et al. (39) We speculate that this discrepancy may stem from differences in disease onset: our GLILD+ cohort exhibited an earlier CVID onset, with six of nine patients manifesting symptoms in early infancy. This persistent immune activation may contribute to the observed lymphocytic expansion, particularly in T-cell subsets, potentially serving as a marker of disease progression. Supporting this, CD8+ central memory T-cell counts became significantly different between GLILD+ and GLILD- groups only at T1, further highlighting its potential as a biomarker for disease worsening.

A further notable finding in our study was the lower mean concentration of DNT cells in the GLILD+ group compared to the controls, reaching statistical significance only at T1. This population of cells is known to have diverse and sometimes contrasting roles in immune dysregulation, including the modulation of inflammation, immune disorders, and cancers (40). DNT cells play a key role in both innate and adaptive immunity, either through TCR-mediated antigen recognition or independent mechanisms. Depending on the immunological milieu, they may function as regulatory, helper, or cytotoxic T cells, influencing adaptive immune responses through direct cell-to-cell interactions. Additionally, DNT cells secrete cytokines such as IL-1, IL-8, IL-10, IL-17, TNF-α, and IFN-γ, which can either facilitate immune homeostasis or exacerbate disease activity in conditions such as autoimmune lymphoproliferative syndrome, Sjögren’s syndrome, systemic lupus erythematosus, and psoriasis (40).

The progressive reduction in DNT cells in the GLILD+ group, reaching significance at T1, may have a dual explanation. On one hand, this decline could be a consequence of the immunomodulatory effects of IgRT, promoting the expansion of both T-regulatory cells and anti-inflammatory DNT cells. On the other hand, it could serve as a surrogate marker of GLILD progression, reflecting a gradual depletion of immunomodulatory T-reg-like DNT cells.

### 4.3 B-cell subset differences between the GLILD+ and GLILD- groups

Regarding the B-cell compartment, the GLILD+ group exhibited significantly lower mean percentages of CD19+ PAN-B, memory, and switched memory B cells, alongside higher percentages of CD19+ naive and CD21low B cells compared to controls. These findings align with the results of Cinetto et al. (29), supporting the hypothesis that CD21low B-cell accumulation represents a consequence of GLILD-associated chronic inflammation and immune activation, which drives immune dysregulation (27). The significant decrease in CD21low B cells at T1 could indicate an IgRT-mediated immunomodulatory effect, reducing autoreactive cell populations.

In terms of serum immunoglobulin concentration at CVID diagnosis, the GLILD+ group exhibited significantly lower IgA and IgG4 count compared to controls, whereas other immunoglobulin classes did not show statistically significant differences. This observation is further supported by a higher prevalence of IgA deficiency in the GLILD+ group (**Table 4**), consistent with prior findings by Cinetto et al., who reported significantly lower IgG and IgA concentrations in GLILD patients compared to CVID controls (29).

### 4.4 Clinical implications and biomarker considerations

Given the widespread knowledge gap among non-immunologist specialists regarding CVID-related immune dysregulation, particularly GLILD, we present a case series illustrating the diverse immune dysregulatory manifestations in patients with CVID. Our findings emphasize the frequent overlap between Chapel’s main clinical phenotypes, reinforcing the concept that CVID is a multifaceted immune dysregulation disorder affecting over 70% of individuals (2, 9, 15).

An interesting observation concerns the mismatch between clinical signs, LFTs, and pulmonary radiological patterns in assessing GLILD progression and response to immunosuppressive therapy. This discordance, particularly noted in the first and second probands, highlights the need for more reliable biomarkers to track disease progression and treatment response, potentially reducing the reliance on radio-imaging, which carries inherent radiation-related risks.

### 4.5 GLILD treatment insights

A summary of GLILD treatment strategies in our cohort is provided in **Tables 5**, **6**.

**Table 5:** A brief focus on GLILD management in our cohort.

Sex	F 7/9 (78%); M 2/9 (22%)
Mean age at last follow-up	48 years
Mean age at CVID diagnosis (SD)	38 years (18 years)
Mean age at GLILD diagnosis (SD)	44 years (15 years)
Patients treated	6/9 (67%)
Patients receiving a single immunosuppressive treatment	2/9 (22%) - 1/9 prednisone (11%) - 1/9 MMF (11%)
Patients receiving subsequent immunosuppressive treatments	4/9 (44%) - 2/9 MMF -> rituximab (22%) - 1/9 prednisone -> MMF -> rituximab (11%) - 1/9 prednisone -> MMF (11%)
Patients with SARS-CoV-2 on MMF therapy	4/4 (100%)
Patients with chronic CMV infection on immunosuppressive therapy	5/6 (83%) - 1/9 prednisone (11%) - 1/9 prednisone -> MMF -> rituximab (11%) - 1/9 MMF (11%) - 2/9 MMF -> rituximab (22%)
Prednisone dosage	- ≥ 0.3mg/kg/day (50mg/day) in 2/3 patients with a successful response - 0.125mg/kg/day (6.25mg/day) in 1/3 patient with no response
Prednisone duration	- 1/3 patient: 3 cycles of 11, 3 and 6 months, respectively - 1/3 patient: 2 cycles of 3 months each - 1/3 patient: continuous low-dose for 7 years
Prednisone efficacy	Clinical-radiological-functional response in 2/3 cases (67%), with relapses at discontinuation
Patients showing prednisone adverse effects	0
MMF dosage	2 g/day
MMF mean duration	26 months (range: 20-30 months)
Patients showing MMF efficacy	4/4 (100%) - 2/4 clinical/functional/radiological response - 2/4 clinical/functional response with radiological progression
Patients showing MMF adverse effects	- 2/5 gastro-intestinal intolerance (diarrhoea/abdominal pain) (40%)
Rituximab scheme	2/3 patients: 375mg/mq/weekly for 4 weeks 1/3 patients: 490mg/mq/dose 2-weeks-apart
Patients showing rituximab efficacy	2/3 (67%) - 1/3 clinical/radiological ineffectiveness and functional steady state - 1/3 clinical/radiological/functional response - 1/3 functional/radiologic improvement with a steady clinical state
Patients showing rituximab adverse effects	0

CVID, common variable immunodeficiency; GLILD, granulomatous lymphocytic interstitial lung disease; MMF, mofetil mycophenolate, SD, standard deviation.

**Table 6:** Treatment criteria in CVID-related GLILD.

Patients (reported according to the case series order)	Treatment criteria				
	Symptomatic disease with poor quality of life	Low DLCO or FVC detected after optimisation of immunoglobulin therapy	Decline in DLCO or FVC during follow up visits	Radiologic progression with fibrotic involvement detected on thoracic HRCT	No treatment needed
1		X		X	
2	X	X		X	
3	X			X	
4		X		X	
5	X			X	
6					X
7	X	X	X	X	
8					X
9					X

CVID, common variable immunodeficiency; DLCO, diffusing lung capacity for carbon monoxide; FVC, forced vital capacity; GLILD, granulomatous lymphocytic interstitial lung disease; HRCT, high-resolution computed tomography.

Our case series provides a novel perspective on CVID-related GLILD treatment, an area lacking robust RCT-derived evidence.

According to the most recent literature on GLILD treatment, Smits et al. found that first-line high-dose steroid monotherapy (≥0.3 mg/kg prednisone equivalent) was superior to a “wait and see” approach in term of HRCT Hartman score and LFTs in an observational cohort study including 39 patients and 20 controls with GLILD, with a 67% response rate and an at-least-2-years-lasting remission in 42% of cases in the absence of major adverse events; though, steroid efficacy in relapses, which were not influenced by low-dose maintenance therapy, was poor (20%) (41).

The importance of this study is corroborated by Bintalib et al., the first to document the incessant drop in lung function in four GLILD patients receiving no immunosuppressive treatment with a mean follow-up of 7.5 years, emphasizing the potential significance of early immunosuppressive intervention when initial tests indicate a potential decline in lung function (42).

In the case of impaired LFTs, high concentrations of CD21low B cells alongside a low count of marginal zone B cells and IgA may serve as additional indicators for initiating immunosuppressive therapy, as these parameters correlated with treatment necessity in a retrospective study on 38 individuals with GLILD by Scarpa et al. (43).

Moreover, Bintalib et al. assessed the long-term efficacy of MMF-based maintenance in four patients with GLILD on steroids, allowing for their gradual discontinuation in 2/4 cases and reduction in the other 2/4 subjects, suggesting that MMF could be considered in the GLILD treatment regimen once a positive response to high-dose steroids is achieved (42).

As concerns alternative immunosuppressants, Tessarin et al. described a case series of six patients with GLILD receiving rituximab as first-line therapy with a scheme of 375 mg/mq/month for six infusions, followed by maintenance every 3 months for a mean duration of 2.4 years: the treated subjects experienced both a functional and radiological improvement, characterized by a statistically significant increase in TLC and DLCO and reduction in the Baumann score, respectively, with no serious adverse events (44).

As for the mammalian target of rapamycin (mTOR) inhibitor sirolimus, it holds potential as a targeted therapy for GLILD. Given the role of mTOR signaling in T-cell activation, differentiation, and inflammatory cytokine production, its inhibition can help reduce excessive immune activation and granuloma formation typical of GLILD. Additionally, sirolimus impacts macrophage function and epithelial lung damage, key contributors to GLILD pathogenesis. So far, anecdotal evidence supports its efficacy, as demonstrated in a case report where sirolimus monotherapy at a dose of 2.5 mg/m2 daily induced GLILD remission in a 12-year-old CVID patient unresponsive to rituximab (45).

Our experience with high-dose steroids (≥ 0.3mg/kg/day) in the second and third probands revealed a prompt but short-lived response following discontinuation during GLILD flares. In contrast, MMF demonstrated a sustained and well-tolerated clinical and functional response in 4/4 cases over a mean duration of 26 months, despite the presence of chronic CMV infection. However, 2/4 patients experienced radiological progression, one on-therapy and one off-therapy, within 6 months. One further patient rapidly discontinued MMF due to significant gastrointestinal intolerance. Rituximab, used as a second- or third-line treatment upon MMF failure, yielded a combined clinical-radiological-functional response in one out of 3 cases and a functional-radiological improvement with stable clinical status in another.

Regarding the interplay between chronic CMV infection and GLILD, Marashi et al. demonstrated that chronic CMV infection significantly contributes to immune dysregulation in patients with CVID by driving a hyperproliferative response dominated by late effector CD8+ T cells, skewed toward proinflammatory cytokines such as IFN-γ and TNF-α (46). This leads to an inverted CD4/CD8 ratio and a dysfunctional immune response, exacerbating inflammatory manifestations, including GLILD. The study found that the increased frequency of late effector CD8+ T cells correlates with chronic inflammation and impaired cytotoxicity in patients with CVID, suggesting that CMV-specific late effector CD8+ T cells may contribute to the development and progression of GLILD. From a clinical perspective, antiviral therapy (e.g., ganciclovir) and anti-TNF-α treatments, such as infliximab, and disease-modifying antirheumatic drugs such as MMF, as used in our patients, may help control CMV replication, reduce inflammation, and improve outcomes in patients with CVID with hyperinflammatory conditions such as GLILD (47).

Among our patients with GLILD, 7/9 experienced SARS-CoV-2 infection with favorable outcomes, even though 4/7 were on MMF therapy. Since 6/7 infections dated back no more than August 2022, this may be attributed to the predominance of the Omicron variant, which is less frequently associated with severe complications (48), along with vaccination coverage (at least three doses in 6/7 cases) and the use of targeted monoclonal antibodies or protease inhibitors, respectively, in 1/7 and 3/7 cases. Our findings align with a recent Italian multicentric study examining COVID-19 outcomes in patients with IEI (49), providing additional insights into SARS-CoV-2 infection management in patients with CVID-GLILD.

Additionally, three untreated GLILD patients exhibited a slight decline in DLCO alongside radiological progression on HRCT, further supporting Bintalib et al.’s recommendation for early immunosuppressive intervention (42).

Treatment employed for GLILD also has a role in other CVID-related immune dysregulation phenomena:

- In immune cytopenias, rituximab and corticosteroids remain first-line treatments, with high-dose immunoglobulins and splenectomy as additional options. Sirolimus shows promise by expanding Tregs and modulating T-helper 1-driven inflammation, while thrombopoietin receptor eltrombopag and anti-proteasome bortezomib are considered in refractory cases.
- For granulomatous disease, including GLILD, rituximab and corticosteroids are commonly used, along with anti-Tumour Necrosis Factor (TNF) agents. Azathioprine, MMF, and soluble cytotoxic T-lymphocyte associated protein 4 (CTLA-4) receptor mimic abatacept may be beneficial in selected cases. Sirolimus, by regulating T-cell activation and macrophage-driven inflammation, represents a promising alternative.
- In enteropathy, corticosteroids, 5-ASA, and anti-TNF agents are standard treatments. Vedolizumab and ustekinumab have been reported effective, while tofacitinib and ustekinumab may offer potential alternatives by targeting the Janus Kinase (JAK) and T-helper-17 pathways, respectively. Gut dysbiosis and endotoxemia could be managed with rifaximin, while larazotide aims to restore mucosal integrity.
- Lymphoproliferation is often treated with rituximab and corticosteroids, but sirolimus and abatacept could modulate T-cell-driven lymphoid hyperplasia (50).

**Table 7** resumes the main steps of GLILD diagnosis, monitoring, and management, with a mention of differential diagnoses (51–54), while **Table 8** summarizes the different lines of treatment for GLILD according to most recent literature.

**Table 7:** Diagnosis, monitoring, and management of GLILD, with a mention of differential diagnosis.

Step	GLILD diagnosis	Differential Diagnosis
1. Initial Clinical & Radiological Evaluation	- Compatible clinical-radiological presentation - HRCT (thin slice, continuous, without contrast) - PFTs including DLCO	- Similar radiological findings may occur in infections (TB, fungal infections, atypical bacterial pneumonia) and malignancies (lymphoma, metastatic lung disease)
2. Histopathological Confirmation	- Presence of granuloma and/or lymphoid hyperplasia on biopsy (surgical open lung/VATS) - Transbronchial biopsy or extra-pulmonary biopsy may be used - BALF analysis to assess cell subpopulations and rule out infections	- Infectious causes may show necrotizing granulomas (TB, fungal infections) - Lymphomas may show atypical lymphoid proliferation
3. Exclusion of Other Diseases	- Exclusion of alternative granulomatous diseases (e.g., sarcoidosis) - No definitive BALF immunophenotype, but high CD21low B cells may support diagnosis - Microbiological investigation (bacteria, mycobacterial, and fungal cultures)	- Sarcoidosis: uniform granulomas with perilymphatic distribution - Hematologic malignancies: CD20+, PAX5+ B cells in lymphoid tissue
4. Imaging Techniques	- HRCT: multiple non-perilymphatic small nodules, ground-glass opacities, low-grade bronchiectasis, mediastinal lymphadenopathy - PET/CT may be used for assessment and monitoring	- Sarcoidosis: perilymphatic nodules, lymph node calcifications - Infections: consolidation with halo signs, necrotizing features
5. Routine Monitoring	- Spirometry and DLCO annually - HRCT every 5 years - Lung MRI under evaluation	- Imaging and lung function tests help differentiate between chronic infection and malignancy
6. Management Approaches	- Asymptomatic: optimization of IgRT - Symptomatic: prednisone (20–40 mg) as first-line - Steroid-refractory: rituximab-azathioprine…(consult **Table 8**)	- Infections: targeted antibiotics (based on BALF culture) - Malignancies: chemotherapy, immunotherapy, radiation

BALF, bronchoalveolar lavage fluid; DLCO, diffusing capacity of the lung for carbon monoxide; GLILD, granulomatous lymphocytic interstitial lung disease; HRCT, high-resolution computed tomography; IgRT, immunoglobulin replacement therapy; MRI, magnetic resonance imaging; PET/CT, positron emission tomography/computed tomography; PFTs, pulmonary function tests; TB, tuberculosis; VATS, video-assisted thoracoscopic surgery.

**Table 8:** A summary of different lines of treatment for CVID-related GLILD.

Line of Treatment	Therapeutic Approach	Dosage	Rationale	Considerations
First-Line	Glucocorticoid Monotherapy	Prednisone 0.3-1 mg/kg/day, taper over 6-12 weeks	Effective in almost 70% of cases, offers rapid immunomodulation, and is affordable	Short-lived response in some cases; side effects include osteoporosis, hyperglycemia, and adrenal suppression
Second-Line	Rituximab Monotherapy	375 mg/m² weekly for 4 weeks (evaluate an every 3 months maintenance) or 1,000 mg IV on days 1 and 15	Targets B-cell dysregulation and pulmonary lymphoproliferation	Risk of progressive multifocal leukoencephalopathy; efficacy supported by limited case series; may be preferred in monotherapy in patients with clinical T-cell deficiency
Third-Line	Rituximab + Antimetabolite (Azathioprine or Mycophenolate Mofetil - MMF)	Rituximab as above + Azathioprine 1-2 mg/kg/day or MMF 500-2,000 mg/day	Combination therapy improves lung function and CT findings	MMF shows sustained response but may cause GI intolerance; relapse possible in some patients
Fourth-Line	Abatacept (CTLA-4-Ig)	10 mg/kg IV on days 1, 15, 30, then every 4 weeks	Effective in CTLA-4 and LRBA deficiencies; modulates T-cell activation and reduces immune dysregulation	Response rate <40% in genetically undetermined CVID cases; lacks broad efficacy outside of monogenic causes
	Sirolimus (mTOR inhibitor)	2.5 mg/m2/day (serum therapeutic range 5-10 ng/ml)	Expands T-regs and modulates T-helper 1-driven and macrophage-driven inflammation	Anecdotal evidence. Sirolimus monotherapy induced GLILD remission in a pediatric patient unresponsive to rituximab
	Disease-Modifying Antirheumatic Drugs (DMARDs) or Other Biologics (Anti-TNF, JAK inhibitors)	Anti-TNF: Infliximab 5 mg/kg every 4-8 weeks or Adalimumab 40 mg every 2 weeks; JAK inhibitors: Tofacitinib 5 mg BID	Targets chronic inflammation and granulomatous progression	Anti-TNF agents may be useful for CMV-associated hyperinflammation; JAK inhibitors show promise but need further study
Fifth-Line	Hematopoietic Stem Cell Transplantation	Individualized based on donor availability and conditioning regimen	Considered in refractory cases	Risk of GLILD recurrence post-transplant, particularly in LRBA deficiency

BID, twice a day; CTLA4, cytotoxic T-lymphocyte associated protein 4; CVID, common variable immunodeficiency; GI, gastro-intestinal; GLILD, granulomatous lymphocytic interstitial lung disease; IV, intravenous; JAK, Janus Kinase; LRBA, lipopolysaccharide responsive beige-like anchor protein; m-TOR, mammalian target of rapamycin; TNF, tumor necrosis factor.

### 4.6 Study limitations

The primary limitations of our study include the small sample size and its retrospective design. Another notable limitation is the absence of systematic radiological assessment using Baumann’s specific score, preventing us from evaluating its reliability as a predictive biomarker for GLILD.

## 5 Conclusions

Our study provides a comprehensive clinical, pulmonary functional, and radiological characterization of a CVID cohort, offering an extensive immunological profile of patients with GLILD, including detailed T- and B-cell subset analyses. We present preliminary data suggesting that immunophenotypic alterations over time may have pragmatic implications for assessing GLILD progression. However, these findings require validation in larger cohorts to determine whether total lymphocyte expansion, along with increased DNT, CD4+, and CD8+ T cells, reliably reflects disease worsening in clinical, functional, and radiological terms.

These insights underscore the urgent need for further research to identify reliable biomarkers for GLILD development and progression. A deeper understanding of the immunological mechanisms underlying multisystemic polyclonal lymphoproliferation, particularly its pulmonary manifestation as GLILD, could inform more targeted management strategies. This may, in turn, reduce reliance on invasive histopathological and radiological assessments, which, despite their risks, remain more practical than clinical and functional markers in evaluating disease progression.

Finally, our case series highlights the complexity of managing patients with CVID-GLILD, particularly in the context of chronic CMV and SARS-CoV-2 infections during immunosuppressive therapy. We hope these findings contribute to future studies aimed at optimizing GLILD treatment strategies.

## Data Availability

The datasets presented in this study can be found in online repositories. The names of the repository/repositories and accession number(s) can be found in the article/**Supplementary Material**.
