# Supplementary material for: Granulomatous lymphocytic interstitial lung disease in common variable immune deficiency: an in-depth clinical, immunological, functional and radiological exploration with a focus on its management, challenged by chronic CMV infection
Source: Front Immunol. 2025 May 15;16:1589052. doi: 10.3389/fimmu.2025.1589052 (PMC12119541; doi:10.3389/fimmu.2025.1589052)
Supplement: Supplementary file 1 [file SupplementaryFile1.docx]

**Laboratory Investigations**
The complete list of variables considered in laboratory included: • Leukocyte formula through Sysmex XN-20; • Extended lymphocyte immune typization [CD3+ (PAN-T), CD3+CD4+ (helper), CD4+CD45RA+CCR7+ (naїve), CD4+CD45RA−CCR7+ (central memory), CD4+CD45RA−CCR7− (effector memory), CD4+CD45RA+CCR7− (terminal effector memory), CD3−CD8+ (cytotoxic), CD8+CD4 5RA+CCR7+ (naїve), CD8+CD45RA−CCR7+ (central memory), CD8+CD45RA−CCR7− (effector memory), CD8+CD45RA+CCR7− (late effector memory), CD4+CD127−CCR7+CD 25++ (regulatory) (T-reg) and TCRαβ+CD3+CD4−CD8− double-negative T cells, CD4+/CD8+ ratio, CD3+γ+δ+ cells, CD56+CD16+CD3− (natural killer) NK cells, CD19+ (PAN-B), CD19+IgD+CD 27− (naїve), CD19+IgD+CD27+ (memory), CD19+IgD−CD27+ (switched memory), CD19+IgM++CD38++ (transitional), CD19+CD21+lCD38− (CD21low) and CD19+IgM−+CD38++ (plasmablast) B cells] through multiparametric flow cytometry; • Serum immunoglobulin concentrations (IgG and subclasses, IgA, IgM and IgE) through an immunoturbidimetric method (Beckman Coulter, Brea, CA, USA). To execute lymphocyte typization, whole blood on EDTA was stained with an apposite antibody cocktail (Becton Dickinson, Franklin Lakes, NJ, USA) at room temperature for 30 minutes. Afterward, red blood was supplemented with 0.5 mL 1 × BD FACS Lysing solution, then washed with PBS and subsequently suspended in PBS. Samples were acquired on a Facs Canto II (BD Bioscience, San Diego, CA, USA) and analysed with Diva software; the analysis included between 10,000 and 50,000 PBMCs for each patient, depending on sample availability.

**Molecular Analysis**
As concerns molecular assessment, the diagnostic gene panel was developed on the Ion Torrent platform (Thermo Fisher Scientific). The custom panel included 46 genes causative for agammaglobulinemia, CVID, and IEIs with immune-dysregulation based on the last update of the ESID classification. The amplicon design covered 99.97% of the 182,523 kb targeted exons with 942 amplicons. For each exon a 6 bp of padding was included. For Ion torrent library preparation and sequencing, ten nanograms of genomic DNA extracted from peripheral blood were used for library preparation. DNA was amplified using the two gene panel primer pools. PCR pools for each sample were combined and indexed using the Ion Xpress Barcode Adapters kit. The amplified libraries were quantified using the qPCR through the Ion Library TaqMan™ Quantitation Kit. All samples were diluted at 10 pM, then amplicon libraries were pooled for emulsion PCR and chip loading using the Ion Chef system, according to manufacturer's instructions. The final pool of samples was sequenced with Ion GeneStudio S5 system using Ion 510 or Ion 520 Chips. As regards Ion torrent bioinformatic analysis, sequencing reads were aligned to hg19 reference genome. Variant calling was performed using Ion Torrent suite software v5.12 or Ion Reporter software v5.10. Called variants were filtered by allele frequencies, selecting variants with MAF <1% annotated with GnomAD database (<https://gnomad.broadinstitute.org/).> Nonsense, frameshift and canonical splicing variants were considered potentially pathogenic, while the other variants were classified according to Varsome database (<https://varsome.com/).> Called variants with the minimum coverage of 20× were analysed by Integrative Genome Viewer and confirmed by sanger sequencing. All regions covered <20× were re-analysed by Sanger sequencing."
